# Supplementary material for: Metabolic reaction fluxes as amplifiers and buffers of risk alleles for coronary artery disease
Source: Mol Syst Biol. 2025 Apr 2;21(6):676–95. doi: 10.1038/s44320-025-00097-2 (PMC12130253; doi:10.1038/s44320-025-00097-2)
Supplement: Supplementary file 1 — Appendix [file 44320_2025_97_MOESM1_ESM.pdf]

# Appendix for

# Metabolic reaction fluxes as amplifiers and buffers of risk alleles for coronary artery disease

Carles Foguet[1,2,3*], Xilin Jiang[1,2,3,4], Scott C. Ritchie[1,2,3,5,6,7], Elodie Persyn[1,2,3], Yu Xu[1,2,3], Chief Ben-Eghan[1,2,3], Henry J. Taylor[2,3,8], Emanuele Di Angelantonio[2,3,5,6,9,10], John Danesh[2,3,5,6,9,11], Adam S. Butterworth[2,3,5,6,9], Samuel A. Lambert[1,2,3,6,12], Michael Inouye[1,2,3,5,6,7*]

1. Cambridge Baker Systems Genomics Initiative, Department of Public Health and Primary Care, University of Cambridge, Cambridge, UK.
2. British Heart Foundation Cardiovascular Epidemiology Unit, Department of Public Health and Primary Care, University of Cambridge, Cambridge, UK.
3. Victor Phillip Dahdaleh Heart and Lung Research Institute, University of Cambridge, Cambridge, UK.
4. Department of Epidemiology, Harvard T.H. Chan School of Public Health, Boston, MA, USA.
5. British Heart Foundation Centre of Research Excellence, University of Cambridge, Cambridge, UK.
6. Health Data Research UK Cambridge, Wellcome Genome Campus and University of Cambridge, Cambridge, UK.
7. Cambridge Baker Systems Genomics Initiative, Baker Heart and Diabetes Institute, Melbourne, VIC, Australia.
8. Center for Precision Health Research, National Human Genome Research Institute, National Institutes of Health, Bethesda, MD, USA
9. National Institute for Health and Care Research Blood and Transplant Research Unit in Donor Health and Behaviour, University of Cambridge, Cambridge, UK.
10. Health Data Science Research Centre, Fondazione Human Technopole, Milan, Italy.
11. Department of Human Genetics, the Wellcome Trust Sanger Institute, Wellcome Trust Genome Campus, Hinxton, UK.
12. European Molecular Biology Laboratory, European Bioinformatics Institute, Wellcome Genome Campus, Hinxton, Cambridge, UK.

* Corresponding authors: cf545@medschl.cam.ac.uk (C.F.) & mi336@cam.ac.uk (M.I.)

## Table of Contents


| Name | Description | Page |
|---|---|---|
| Appendix Table S1 | Cases and controls of coronary atherosclerosis and myocardial infarction. | 1 |
| Appendix Figure S1 | Representative examples of risk allele dosage-specific reaction effects. | 1 |
| Appendix Figure S2 | Robustness of interaction estimates to the inclusion of additional cardiometabolic covariates. | 2 |
| Appendix Figure S3 | Comparison of interaction effect size and dosage-specific tests. | 3 |
| Appendix Figure S4 | Variants and reaction fluxes with significant interaction. | 3 |
| Appendix Figure S5 | Linkage disequilibrium between eQTL variants used as input for flux estimation and CAD risk variants. | 4 |
| Appendix Figure S6 | Significant SNP-Flux Interactions in the LPA/PLG risk loci. | 5 |
| Appendix Figure S7 | Effect sizes for the risk of coronary atherosclerosis and myocardial infarction. | 6 |

**Appendix Table S1**: Cases and controls of coronary atherosclerosis and myocardial infarction in the inferred European ancestry subset of UKB. Under the definition of coronary atherosclerosis, individuals with any of the constitutive ICD codes of ischemic heart disease were excluded from the controls.

| | | Myocardial Infarction | | |
|---|---|---|---|---|
| | | Control | Case | Excluded |
| Coronary Atherosclerosis | Control | 397254 | 970 | 58 |
| | Case | 10220 | 27682 | 39 |
| | Excluded | 16155 | 7355 | 0 |

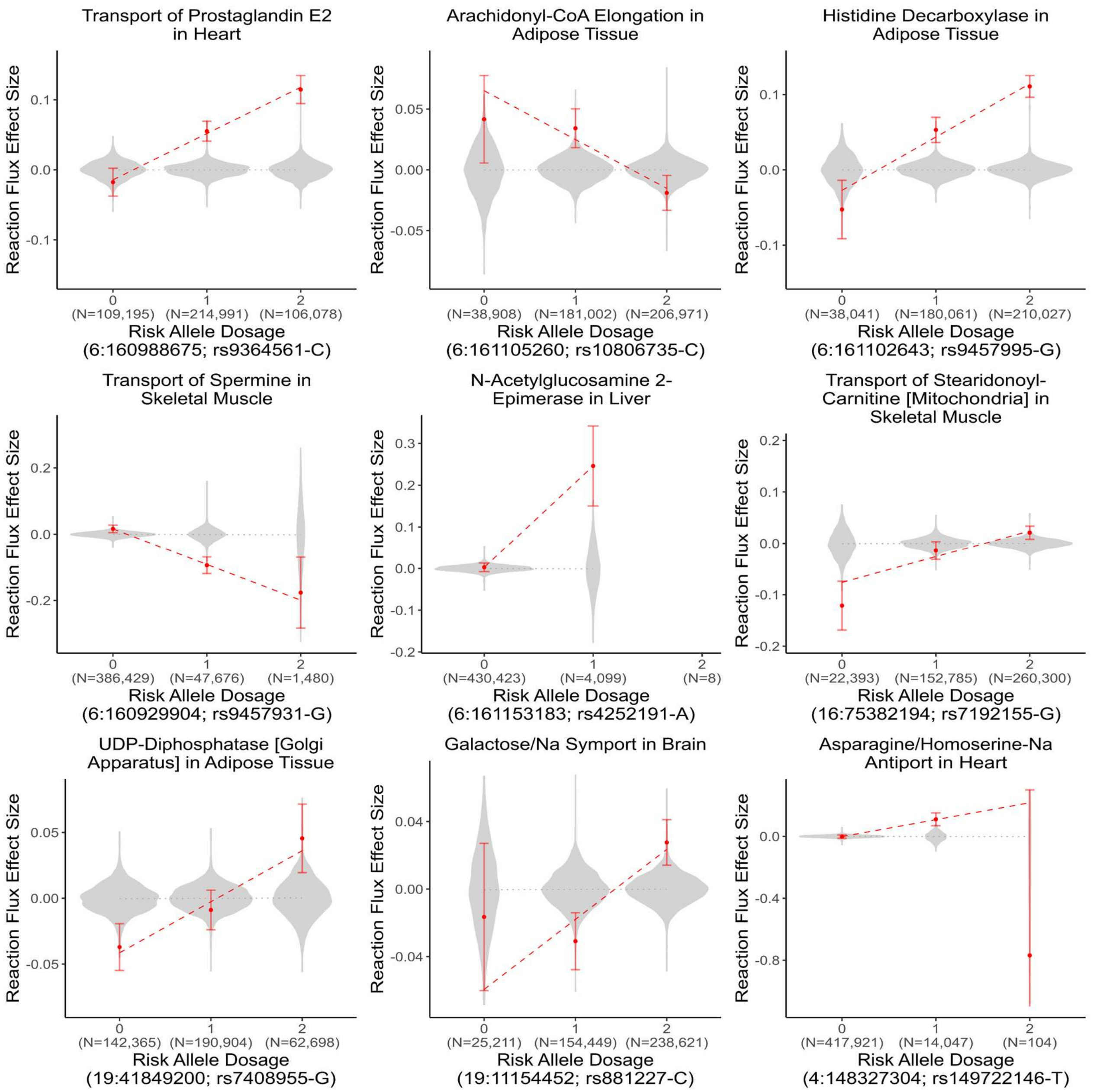


**Appendix Figure S1**: Representative examples of risk allele dosage-specific reaction effects for a set of SNP-reaction flux pairs with significant interaction on CAD risk. Reaction flux effect sizes on CAD risk were estimated using Cox regression in analysed UKB participants carrying different risk allele dosages. Effect sizes are expressed as log(Hazard ratio) per s.d of log(flux value). Error bars denote the 95% confidence intervals

for reaction effect size estimates. The dashed red line indicates the linear regression of flux effect size per dosage weighted by the standard error of effect size estimates (1/$SE^2$). Violin plots indicate the distribution of all other risk reaction flux effect sizes for the same risk allele and the dotted line is the linear regression for these effect sizes. Genome coordinates correspond to the GRCh37 genome assembly.

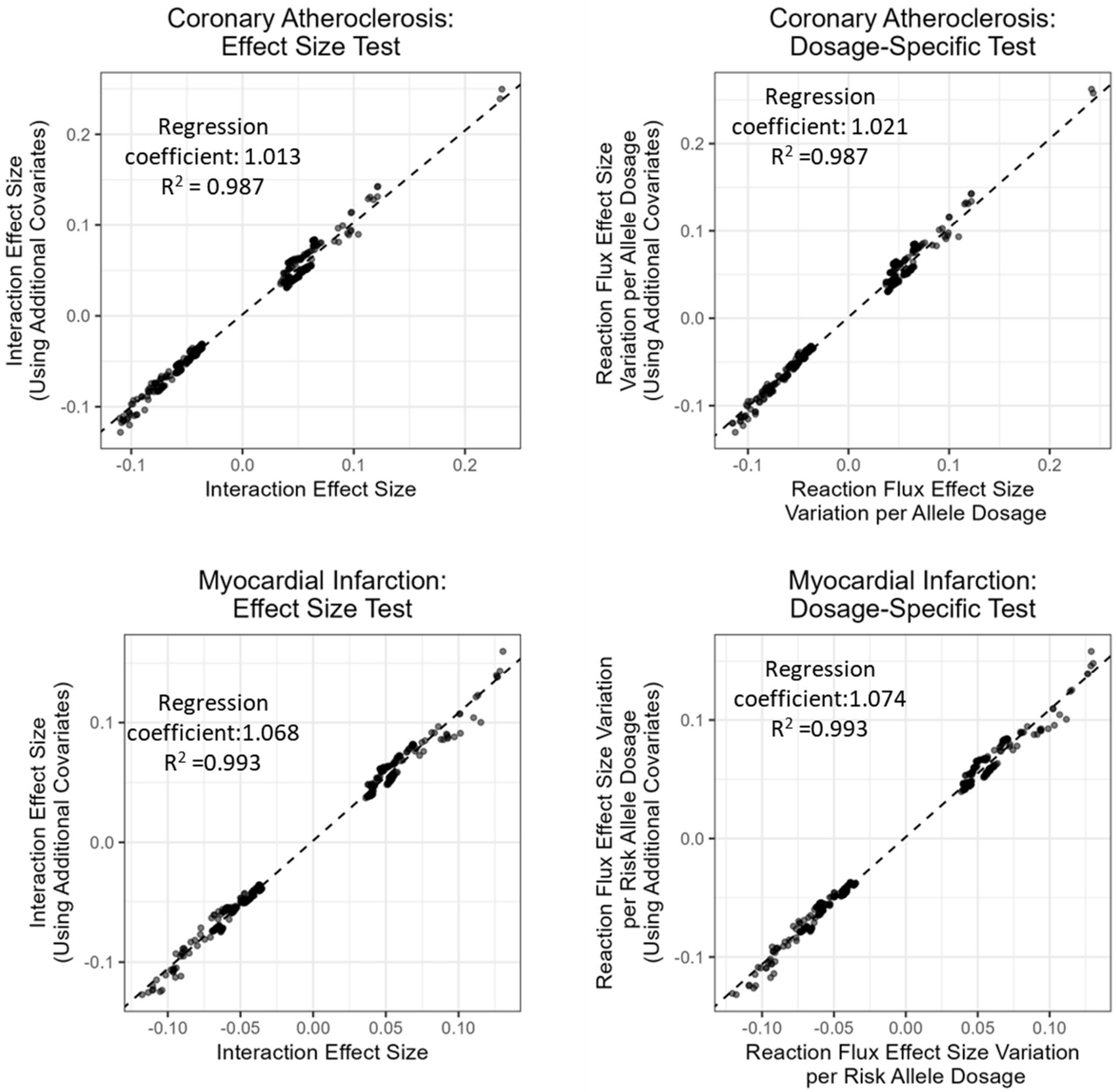


**Appendix Figure S2**: Robustness of interaction estimates to the inclusion of additional cardiometabolic covariates. Pairs of SNPs and reaction fluxes with significant interaction on coronary atherosclerosis or myocardial infarction risk were re-evaluated for interaction accounting for additional cardiometabolic measures (i.e., BMI, systolic blood pressure, and blood levels of LDL-cholesterol, HDL-cholesterol, and triglycerides) as covariates. Interaction effects were quantified using both the interaction effect size test and the dosage-specific test (Methods). The dashed line indicates the linear regression of interaction effects estimates with the additional covariates relative to the original estimates.

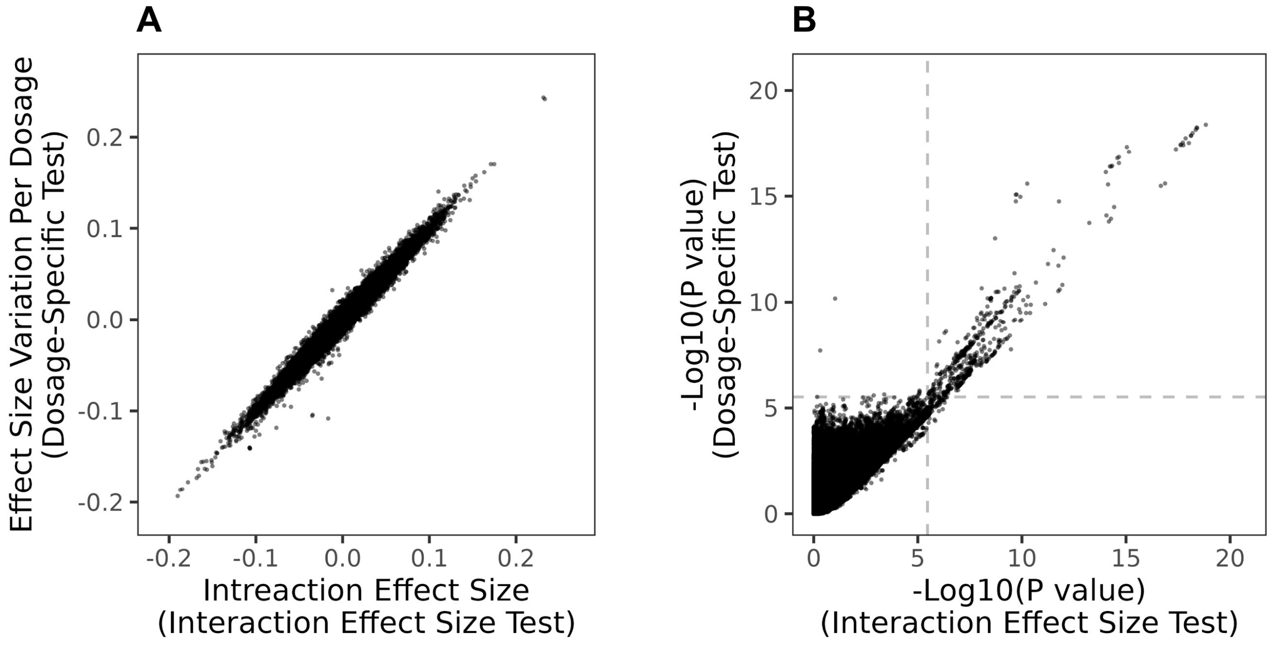


**Appendix Figure S3**: Comparison of interaction effect size and dosage-specific tests. **(A)** Comparison of interaction effect size estimates. **(B)** Comparison of interaction effect significance (-log10(P-values)). The dashed grey line indicates the nominal P-value significance threshold estimated after Benjamini-Hochberg multiple testing correction (i.e., FDR<0.05).

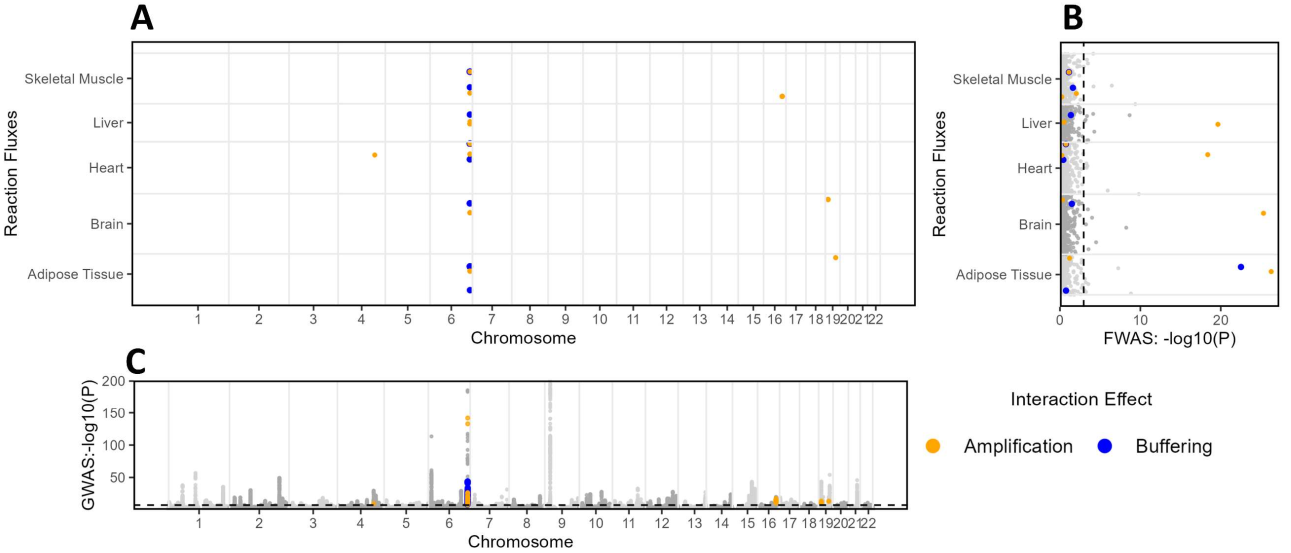


**Appendix Figure S4**: Variants and reaction fluxes with significant interaction. (**A**) Organ metabolic network and chromosome of the reaction fluxes and variants, respectively, with significant interaction on CAD risk. **(B)** and **(C)** Manhattan plots of reaction fluxes and variants effects on CAD risk when analysed in univariate analysis. Variant effect size P-values were obtained from a published GWAS meta-analysis (GCST90132314). P-values for reaction effect size were computed for uncorrelated reaction fluxes with a Cox proportional hazard model in the analysed subset of UKB. The dashed grey line indicates the P-value threshold to consider a variant or reaction effect size significant.

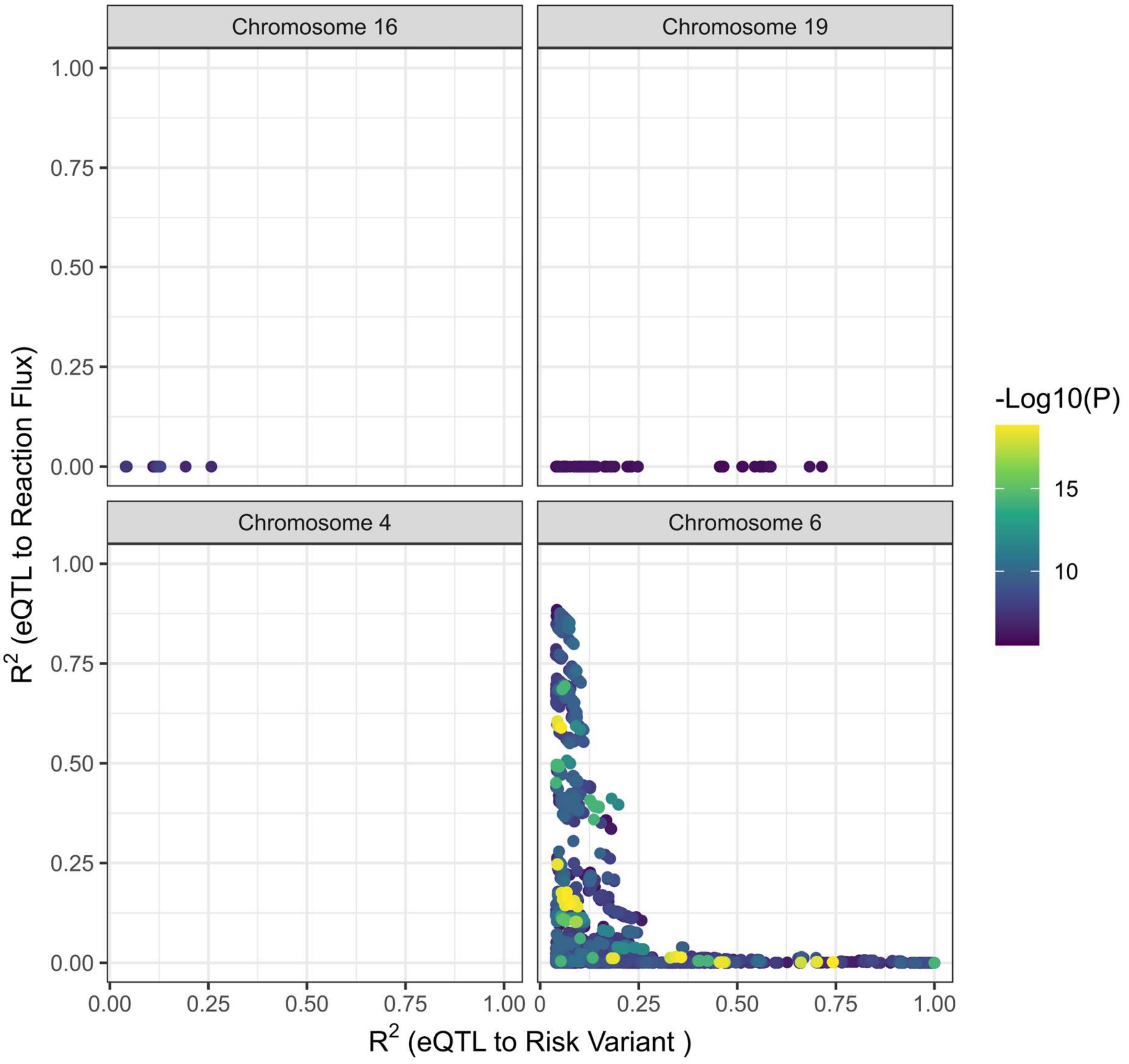


**Appendix Figure S5:** Linkage disequilibrium between eQTL variants used as input for flux estimation and CAD risk variants with significant amplification and buffering effects. For those eQTL variants with linkage disequilibrium above 0.04, their correlation to fluxes significantly amplifying or buffering the risk variants is plotted. As outlined in methods, for each pair of reaction flux and CAD risk variant evaluated for interaction, the effect of the latter was regressed out of the flux. Data is coloured based on the interaction effect size P-value for each risk-variant reaction flux pair.

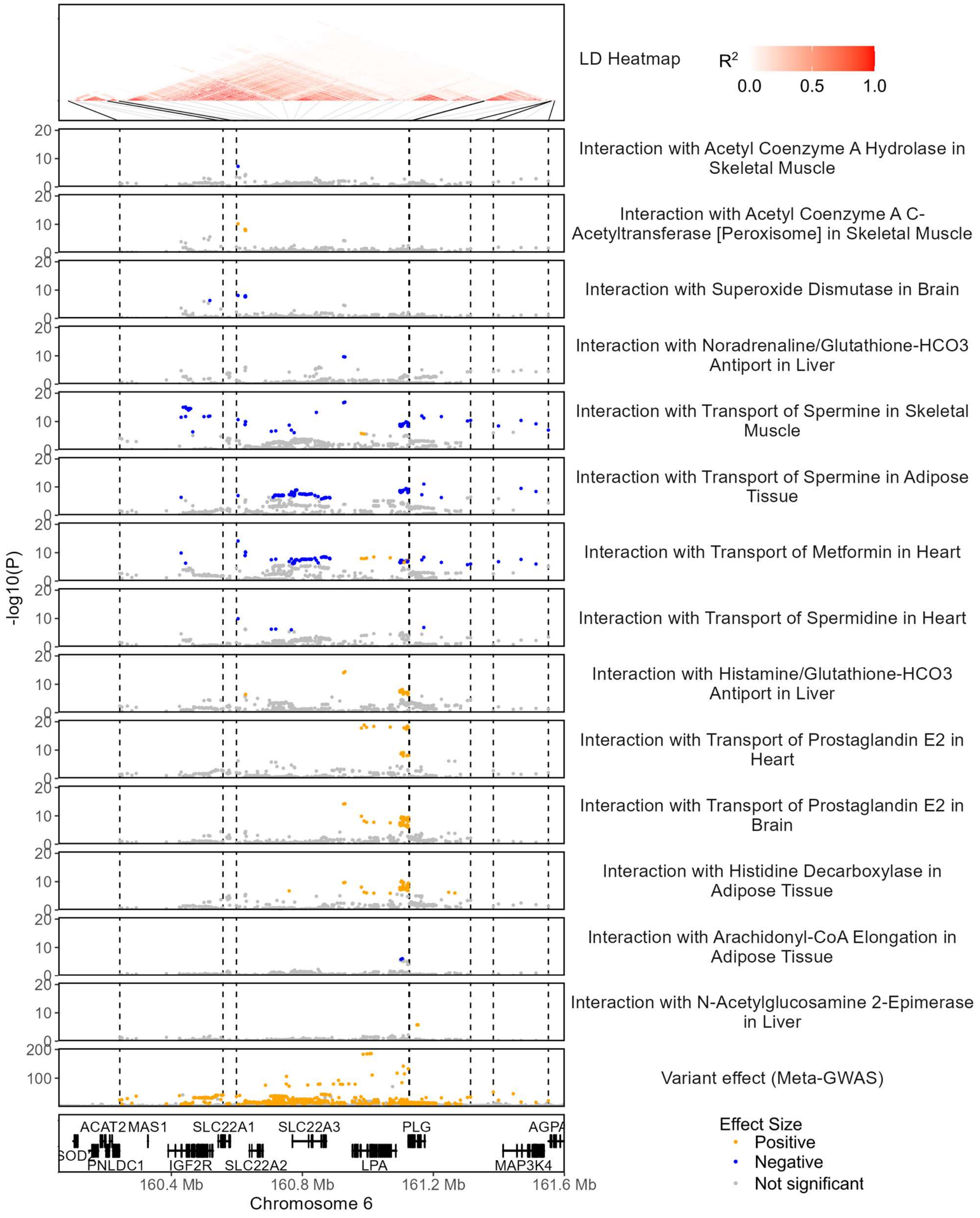


**Appendix Figure S6**: Significant SNP-Flux Interactions in the LPA/PLG risk loci. The regional association plots show the -log10(P value) for interaction and variant effect sizes on CAD risk. P-values for variant effect sizes were obtained from the meta-GWAS summary statistics (GCST90132314). The LD heatmap indicates the pairwise LD for SNPs with genome-wide significant effect size on CAD in the analysed UKB participants. Dashed black lines indicate the limits of LD blocks ($R^2$>0.6) used to define independent risk loci. To facilitate visualization, only LD blocks with variants involved in significant interactions are highlighted. Only protein-coding genes are shown in the gene plot. Genome coordinates correspond to the GRCh37 genome assembly.

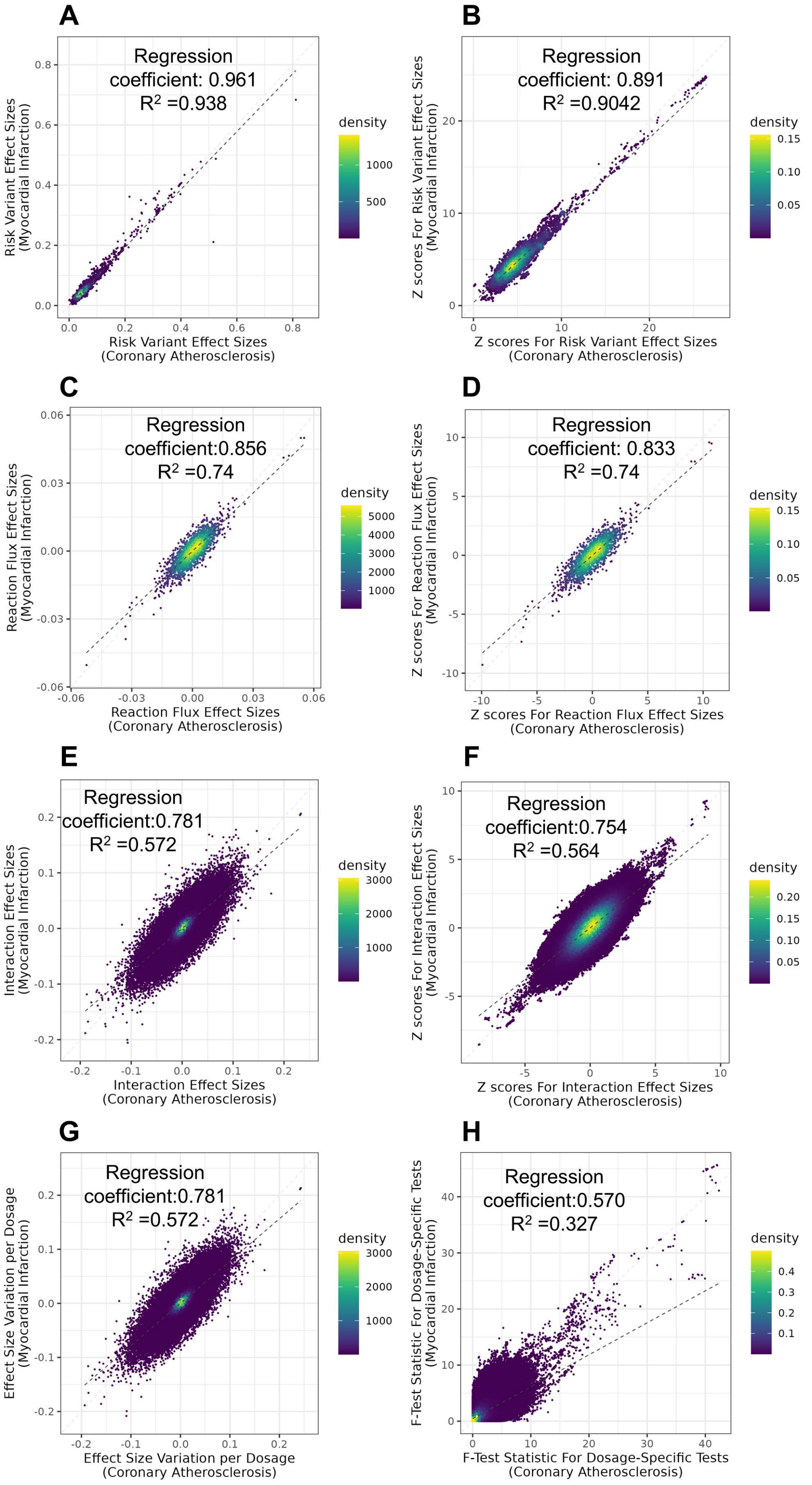
A
Regression
coefficient: 0.961
R2 =0.938
Risk Variant Effect Sizes
(Myocardial Infarction)
Risk Variant Effect Sizes
(Coronary Atherosclerosis)
density
B
Regression
coefficient: 0.891
R2 =0.9042
Z scores For Risk Variant Effect Sizes
(Myocardial Infarction)
Z scores For Risk Variant Effect Sizes
(Coronary Atherosclerosis)
density
C
Regression
coefficient:0.856
R2 =0.74
Reaction Flux Effect Sizes
(Myocardial Infarction)
Reaction Flux Effect Sizes
(Coronary Atherosclerosis)
density
D
Regression
coefficient: 0.833
R2 =0.74
Z scores For Reaction Flux Effect Sizes
(Myocardial Infarction)
Z scores For Reaction Flux Effect Sizes
(Coronary Atherosclerosis)
density
E
Regression
coefficient:0.781
R2 =0.572
Interaction Effect Sizes
(Myocardial Infarction)
Interaction Effect Sizes
(Coronary Atherosclerosis)
density
F
Regression
coefficient:0.754
R2 =0.564
Z scores For Interaction Effect Sizes
(Myocardial Infarction)
Z scores For Interaction Effect Sizes
(Coronary Atherosclerosis)
density
G
Regression
coefficient:0.781
R2 =0.572
Effect Size Variation per Dosage
(Myocardial Infarction)
Effect Size Variation per Dosage
(Coronary Atherosclerosis)
density
H
Regression
coefficient:0.570
R2 =0.327
F-Test Statistic For Dosage-Specific Tests
(Myocardial Infarction)
F-Test Statistic For Dosage-Specific Tests
(Coronary Atherosclerosis)
density

**Appendix Figure S7:** Effect sizes for the risk of coronary atherosclerosis and myocardial infarction. Effect sizes and Z scores for effect sizes for risk variants (**A,B**), reaction fluxes (**C,B**), interaction between reaction fluxes and risk variants (**E,F**), and reaction effect size variation and F-statistic for the dosage-specific test (**G,H**) using two alternative disease definitions: coronary atherosclerosis and myocardial infarction. The set of evaluated risk variants was derived from a published GWAS meta-analysis (GCST90132314). The reaction flux effect size was evaluated for 1,670 uncorrelated reaction fluxes (**Methods**). Effect sizes were evaluated with a Cox proportional hazard model. The dosage-specific test evaluated the variation of reaction flux effect size per each risk allele dosage (**Methods**). The dashed black line indicates the linear regression for the plotted values.
