# Supplementary material for: Metabolic reaction fluxes as amplifiers and buffers of risk alleles for coronary artery disease
Source: Mol Syst Biol. 2025 Apr 2;21(6):676–95. doi: 10.1038/s44320-025-00097-2 (PMC12130253; doi:10.1038/s44320-025-00097-2)
Supplement: Supplementary file 5 — Expanded View Figures [file 44320_2025_97_MOESM5_ESM.pdf]

Expanded View Figures

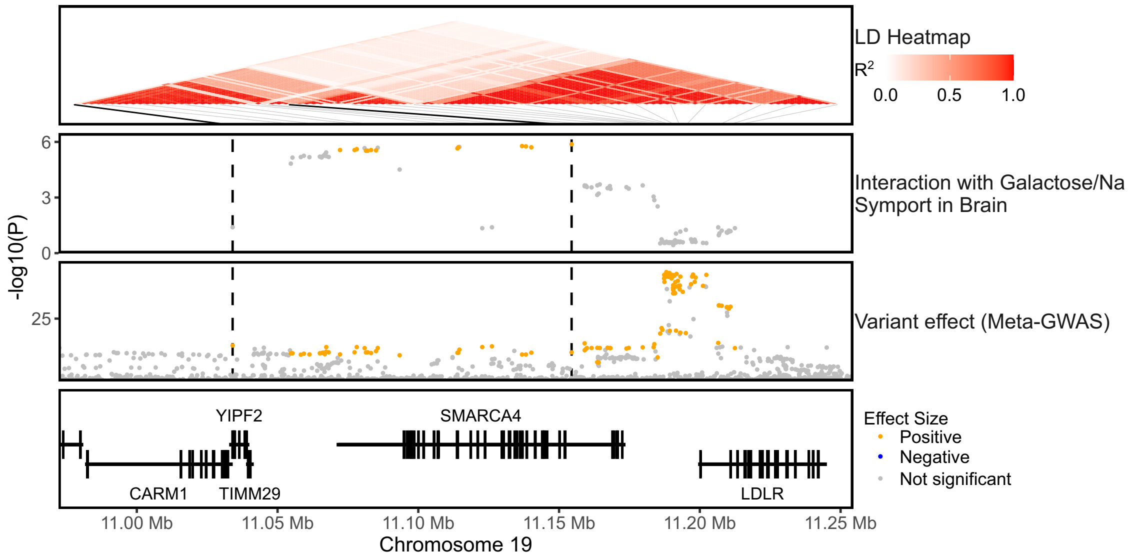

Figure EV1. Interaction between galactose transport in brain and variants in the *SMARCA4* risk locus.

The regional association plots show the −log10(*P*-value) for interaction and variant effect sizes on CAD risk.

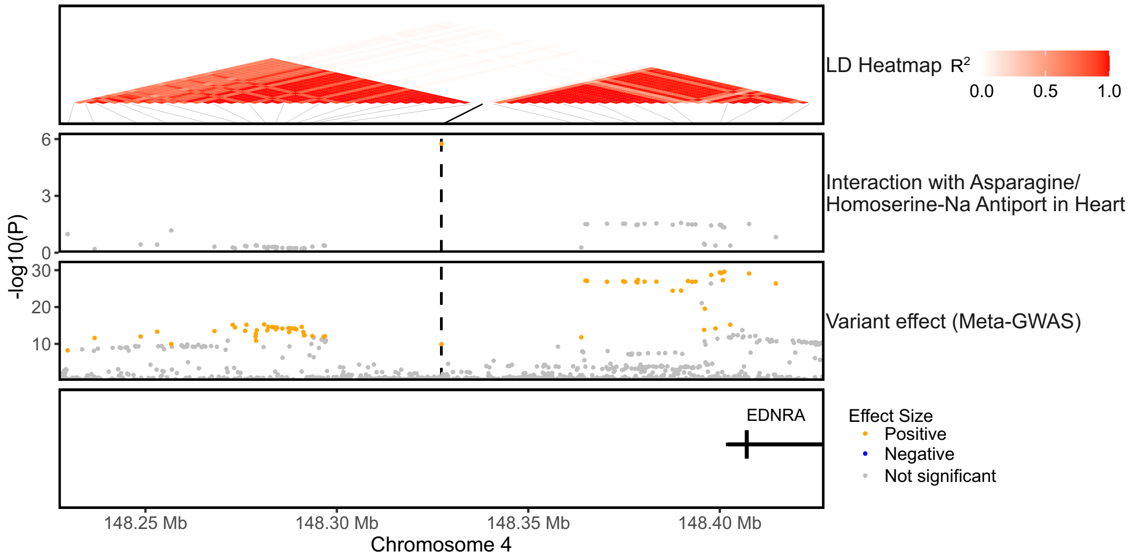

Figure EV2. Amino acid transport amplifies the effect of a risk variant at the *EDNRA* locus.

The regional association plots show the −log10(*P*-value) for interaction and variant effect sizes on CAD risk.

© The Author(s)

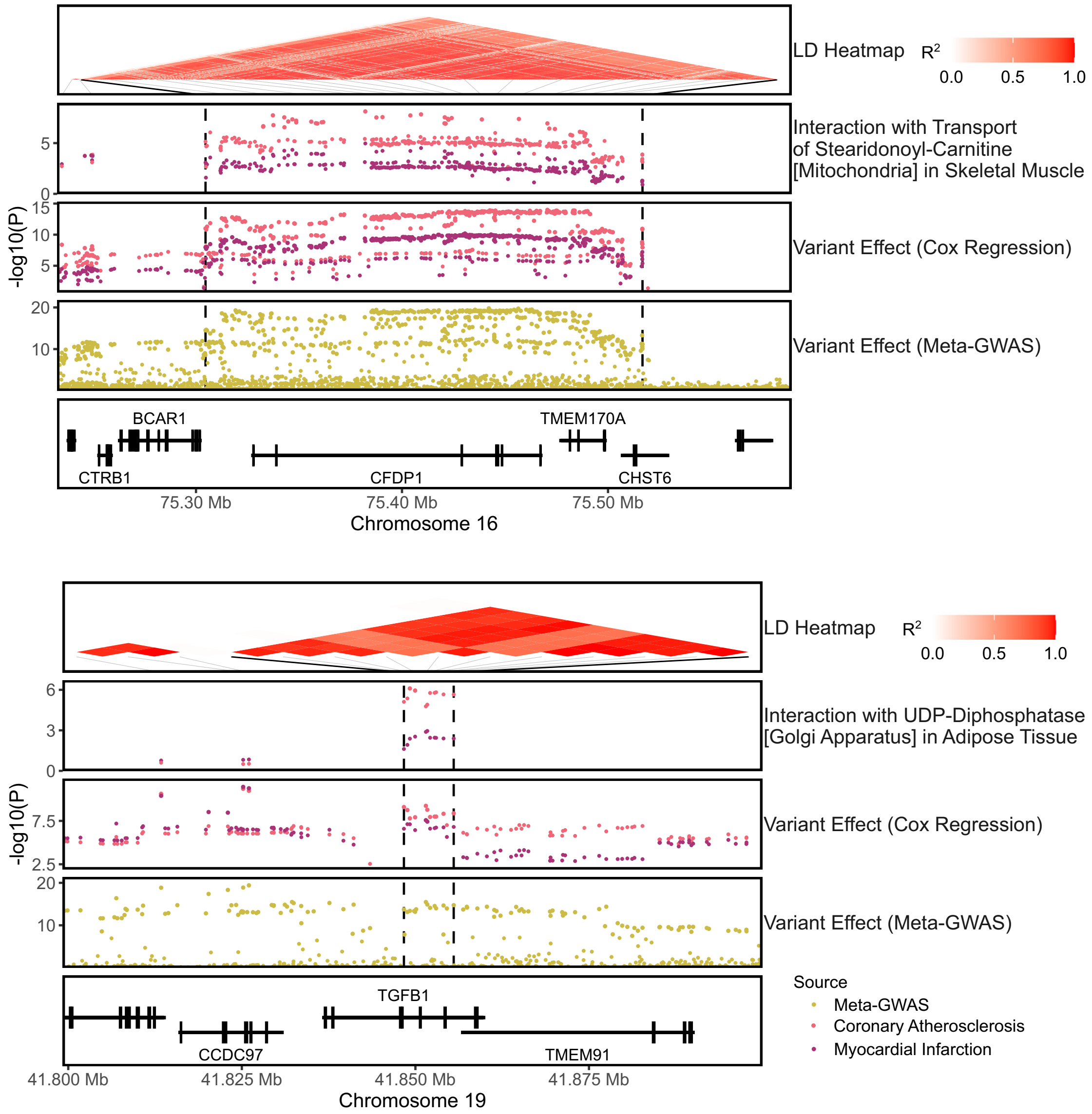

Figure EV3. Example of interactions that are attenuated in myocardial infarction.

The regional association plots show the −log10(*P*-value) for interaction and variant effect sizes on disease risk. *P*-values for interaction and variant effects (Cox Regression) were derived from the interaction and variant effect size tests, respectively, for coronary atherosclerosis or myocardial infarction events. As a reference, the variant effects from the meta-GWAS on CAD risk (Aragam et al, 2022) are also plotted.

© The Author(s)

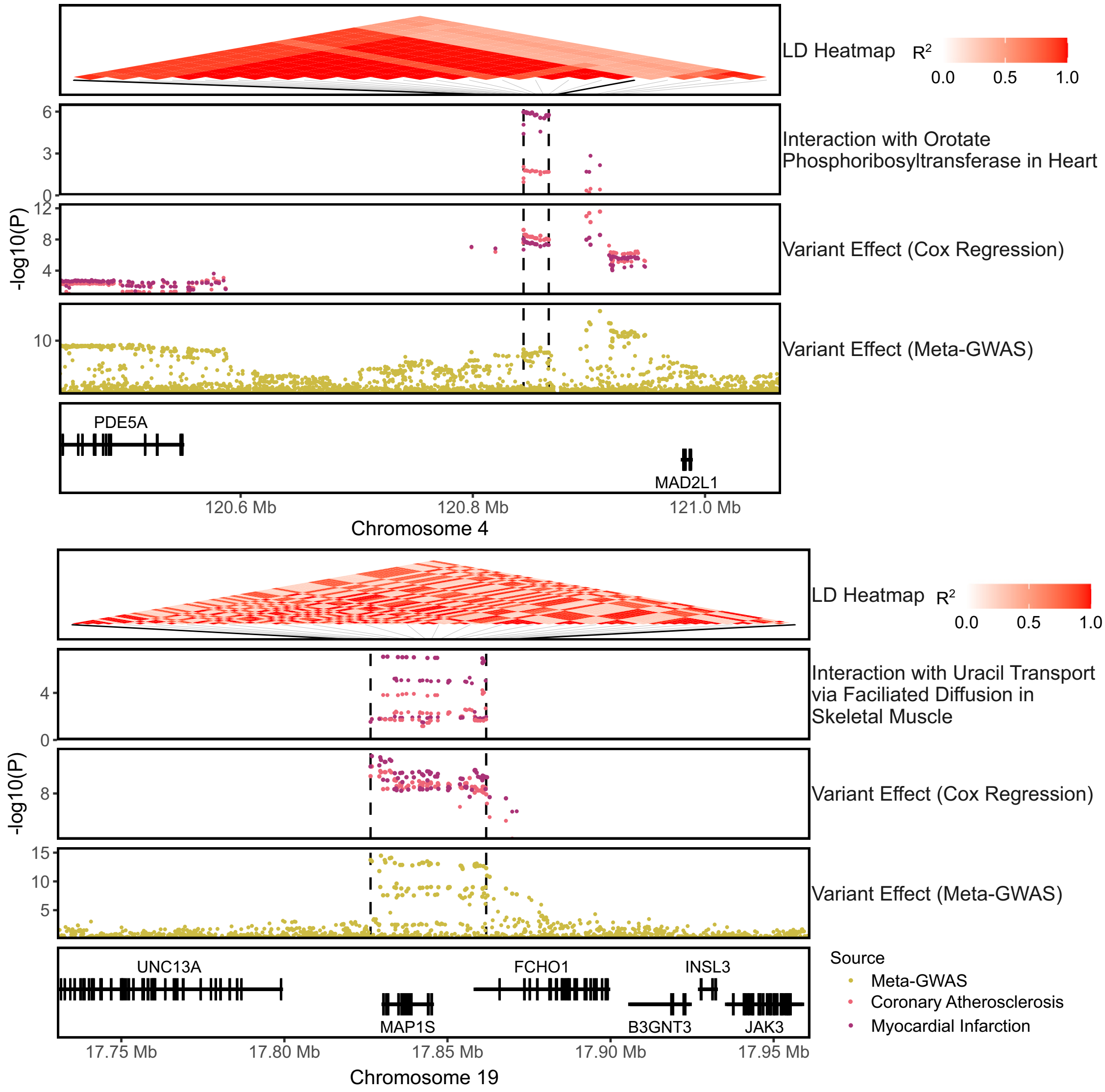

Figure EV4. Example of interactions that are specific to myocardial infarction.

The regional association plots show the −log10(*P*-value) for interaction and variant effect sizes on disease risk. *P*-values for interaction and variant effects (Cox Regression) were derived from the interaction and variant effect size tests, respectively, for coronary atherosclerosis or myocardial infarction events. As a reference, the variant effects from the meta-GWAS on CAD risk (Aragam et al, 2022) are also plotted.
